# A novel lysosome-related gene signature coupled with gleason score for prognosis prediction in prostate cancer

**DOI:** 10.3389/fgene.2023.1135365

**Published:** 2023-03-30

**Authors:** Ying Huang, Fan Yang, Wenyi Zhang, Yupeng Zhou, Dengyi Duan, Shuang Liu, Jianmin Li, Yang Zhao

**Affiliations:** ^1^ Department of Radiology, The Second Hospital of Tianjin Medical University, Tianjin, China; ^2^ Tianjin Institute of Urology, The Second Hospital of Tianjin Medical University, Tianjin, China; ^3^ Department of Urology, The Second Hospital of Tianjin Medical University, Tianjin, China

**Keywords:** prostate cancer, lysosome, prognosis, gene signature, gleason score

## Abstract

**Background:** Prostate cancer (PCa) is highly heterogeneous, which makes it difficult to precisely distinguish the clinical stages and histological grades of tumor lesions, thereby leading to large amounts of under- and over-treatment. Thus, we expect the development of novel prediction approaches for the prevention of inadequate therapies. The emerging evidence demonstrates the pivotal role of lysosome-related mechanisms in the prognosis of PCa. In this study, we aimed to identify a lysosome-related prognostic predictor in PCa for future therapies.

**Methods:** The PCa samples involved in this study were gathered from The Cancer Genome Atlas database (TCGA) (*n* = 552) and cBioPortal database (*n* = 82). During screening, we categorized PCa patients into two immune groups based on median ssGSEA scores. Then, the Gleason score and lysosome-related genes were included and screened out by using a univariate Cox regression analysis and the least absolute shrinkage and selection operation (LASSO) analysis. Following further analysis, the probability of progression free interval (PFI) was modeled by using unadjusted Kaplan–Meier estimation curves and a multivariable Cox regression analysis. A receiver operating characteristic (ROC) curve, nomogram and calibration curve were used to examine the predictive value of this model in discriminating progression events from non-events. The model was trained and repeatedly validated by creating a training set (*n* = 400), an internal validation set (*n* = 100) and an external validation (*n* = 82) from the cohort.

**Results:** Following grouping by ssGSEA score, the Gleason score and two LRGs—neutrophil cytosolic factor 1 (NCF1) and gamma-interferon-inducible lysosomal thiol reductase (IFI30)—were screened out to differentiate patients with or without progression (1-year AUC = 0.787; 3-year AUC = 0.798; 5-year AUC = 0.772; 10-year AUC = 0.832). Patients with a higher risk showed poorer outcomes (*p* < 0.0001) and a higher cumulative hazard (*p* < 0.0001). Besides this, our risk model combined LRGs with the Gleason score and presented a more accurate prediction of PCa prognosis than the Gleason score alone. In three validation sets, our model still achieved high prediction rates.

**Conclusion:** In conclusion, this novel lysosome-related gene signature, coupled with the Gleason score, works well in PCa for prognosis prediction.

## 1 Introduction

Prostate cancer (PCa) has been ranked first among newly diagnosed cancers in men globally. Although early stage, primary PCa cases can be treated effectively by surgery or radiation therapy, a considerable portion of these patients unavoidably progress to advanced PCa with a poor prognosis (Taitt, 2018; Siegel et al., 2022). Therefore, it is vital to predict the probability of PCa progression in order to guide treatment. The Gleason score, a PCa grading system based on histological patterns of biopsy specimens, has been widely applied for evaluating PCa aggressiveness, predicting prognosis and directing treatment (Gleason and Mellinger, 1974; Epstein et al., 2005; 2016). However, the Gleason score system has exhibited limitations upon distinguishing some aggressive tumors from indolent tumors because of the strong heterogeneity in PCa, resulting in over-treatment or inadequate therapy in clinics (Siegel et al., 2021). Consequently, a novel and robust predictive model is urgently needed for prognostic stratification for the purpose of guiding treatment and ameliorating the outcome of PCa.

As key compartments of cellular homeostasis, lysosomes have been found to be involved in multiple cellular processes including cell death, immune response, energy metabolism, cell signaling and endocytic receptor recycling (Hanahan and Weinberg, 2011; Appelqvist et al., 2013). Recent studies have identified that some lysosome-associated mechanisms play pivotal roles in PCa development and are closely related to PCa prognosis (Zhang et al., 2014; Blessing et al., 2017; Zhu et al., 2022). Although lysosome-related genes (LRGs) may be promising biomarkers for the prediction of a PCa prognosis, few studies have concentrated on building LRGs-based prognosis models.

Studies have increasingly shown that the immune microenvironment plays a crucial role in carcinogenesis and the development of cancer (Quail and Joyce, 2013; Zhang et al., 2013). A strategy using immune scores can refine or even be superior to the cancer staging system, based on the traditional TNM staging system, to further assess overall prognosis (Pagès et al., 2018; Angell et al., 2020). Interestingly, some studies have examined immune cell densities in association with the prognosis of PCa. For example, high levels of regulatory T cells, M1 macrophages and M2 macrophage infiltration were associated with biochemical recurrence (Chang et al., 2022; Choi et al., 2022; Sfanos, 2022). Thus, novel approaches to using an immune scoring system for PCa stratification will provide new insights into prognostic prediction.

To date, lysosomes have been found to influence innate and adaptive immune responses of the body against tumor cells, serving as a regulator in various immune cells, including dendritic cells (DCs), macrophages and T cells (Zhang et al., 2021). A type of secreted lysosome derived from tumor cells particularly dampened the intra-tumoral infiltration of DCs, which undergo apoptosis due to uptake of secreted lysosomes (Santana-Magal et al., 2020). A subset of DCs expressing lysosomal-associated membrane protein 3 (LAMP3) was identified as having the ability to steer effector T cells to migrate into tumors, and showed a favorable prognosis (Zhang et al., 2019).

As noted above, lysosome-related pathways and tumor stratification are of paramount importance in PCa. Given the conclusions described in previous studies, that inclusion of the Gleason score sum with a gene signature can improve the accuracy and sensitivity of a PCa prognostic model (Chen et al., 2012; Corradi et al., 2021), we screened differential expressed lysosome-related genes between the high and low-immune groups in PCa, and established a novel lysosome-related signature coupled with Gleason score to forecast PCa patients’ prognoses, which may provide a robust treatment option and predictive tool for PCa.

## 2 Materials and methods

### 2.1 Data acquisition

We downloaded the transcriptome data (read counts and transcripts per million (TPM)) and corresponding clinical data of PCa from the TCGA database using the R package “TCGAbiolinks”, and randomly divided the tumor data (*n* = 500) into five parts using R package “Caret” and setting seed as “1”, one of which was the internal validation set (*n* = 100) and the rest (*n* = 400) the training set. The external validation set EGAS00001002923 (Gerhauser et al., 2018) with RNA-seq expression (reads per kilobase per million mapped reads (RPKM)) and corresponding clinical data were obtained through the cBioPortal (Prostate Cancer (DKFZ, Cancer Cell 2018)). Data had to meet the following inclusion criteria at the same time: the availability of RNA-seq sequencing data, clinical progression-free survival data and BCR-related indicators. In the aggregate, 552 TCGA-PRAD samples across 52 normal tissues and 500 prostate cancer tissues, of which 400 were a training set and 100 were an internal validation set, as well as 82 samples from EGAS00001002923 used as an external validation set, were collected for analysis. Furthermore, the gene signatures of 28 tumor-infiltrating lymphocytes were captured from the TISIDB database (http://cis.hku.hk/TISIDB/), listed in Supplementary Table S1. A total of 797 LRGs from 23 lysosome-related pathways were obtained from the Molecular Signatures Database, listed in Supplementary Table S2.

### 2.2 Assessment of tumor immune cell infiltration

To compare the composition and differences among 28 kinds of tumor-infiltrating immune cells between cancer samples and normal samples from TCGA-PRAD, we quantified the ssGSEA scores by using an ssGSEA algorithm and R package “GSVA”. Of note, the ssGSEA scores were normalized to a 0–1 interval and processed by the mean value. RNA expression was expressed as TPM.

### 2.3 Immune-related differentially expressed gene (IRG) screening and enrichment analysis

PCa samples from the TCGA database were divided into high-immune and low-immune groups based on the median ssGSEA score. Next, R package “DESeq2” was used to screen out differentially expressed genes (DEGs) between two immune groups; 1,093 IRGs with the adjusted *p*-value < 0.05 and the absolute value of Log2FoldChange >1 was considered as statistically significant and were visualized by a volcano plot. After that, a gene ontology (GO) enrichment analysis and its visualization were applied for IRGs by using R packages “topGO” and “clusterProfiler”. RNA expression was expressed as read counts.

### 2.4 Lysosome-related IRG (LIRG) screening

We generated a Venn diagram to show LIRGs, which were categorized as the shared gene between IRGs and LRGs. Next, we performed a heat map to compare levels of LIRGs between low and high-immune groups. In order to explore the correlation of individual LIRGs and PCa prognosis, the patient progression-free interval (PFI) was determined by the Kaplan–Meier (KM) method and Log-rank test between the high- and low-expression groups based on the scanning cut points with R package “survival” and “survminer”.

### 2.5 Construction and validation of the prognostic model

A univariate Cox regression analysis was used to screen out progression-associated factors among the training set and was shown in a forest plot using R package “forestplot”. Moreover, we used the least absolute shrinkage and selector operation (LASSO) algorithm to further narrow down candidate factors. Then, a multivariate Cox regression analysis was performed to select the factor with independent prognostic value and determine the coefficients of a linear risk score formula. The linear risk score formula of the whole PCa can be calculated as follows:

$$Risk\;score=\sum_{i=1}^{n}\left(Xi\times \beta i\right)$$

In this equation, n represents the number of prognostic factors, X represents the expression of genes or the Gleason score and *ß* represents the regression coefficients of prognostic factors. The proportional hazards (PH) assumption can be checked using R package “survival”. Meanwhile, the training set was grouped into high and low-risk groups based on the cut point of the risk score using R package “timeROC”. A KM curve by log-rank test and a time-dependent receiver operating characteristic (ROC) curve were performed to evaluate the prognostic ability and performance of the prognostic model. According to the results of the multivariate analysis, we applied R package “rms” to establish a nomogram for visualizing the model, which contributes to the guideline of clinical decision-making. The area under the curve (AUC) by R package “timeROC”, the concordance index (C-index) and the calibration curve by R package “rms” were used to assess the model’s predictive accuracy. Furthermore, the performance of the model was verified in the internal and external validation set using the same methods. In the external validation set, we used biochemical recurrence (BCR) to represent progression. The RNA-seq expression mentioned above was processed by Log_2_ (TPM+1).

### 2.6 Statistical analysis

All statistical analyses were performed under R environment (R version 4.2.1). The unpaired two-sample Wilcoxon test (also known as the Mann–Whitney test) was utilized to compare two independent groups. Comparison of categorical variables was evaluated by Fisher’s exact test. R packages “ggplot2”, “ggpubr” and “pheatmap” were applied for different plots. *p* < 0.05 was set as statistically significant in our study.

## 3 Results

### 3.1 Landscape of the tumor immune microenvironment in prostate cancer

**FIGURE 1:**
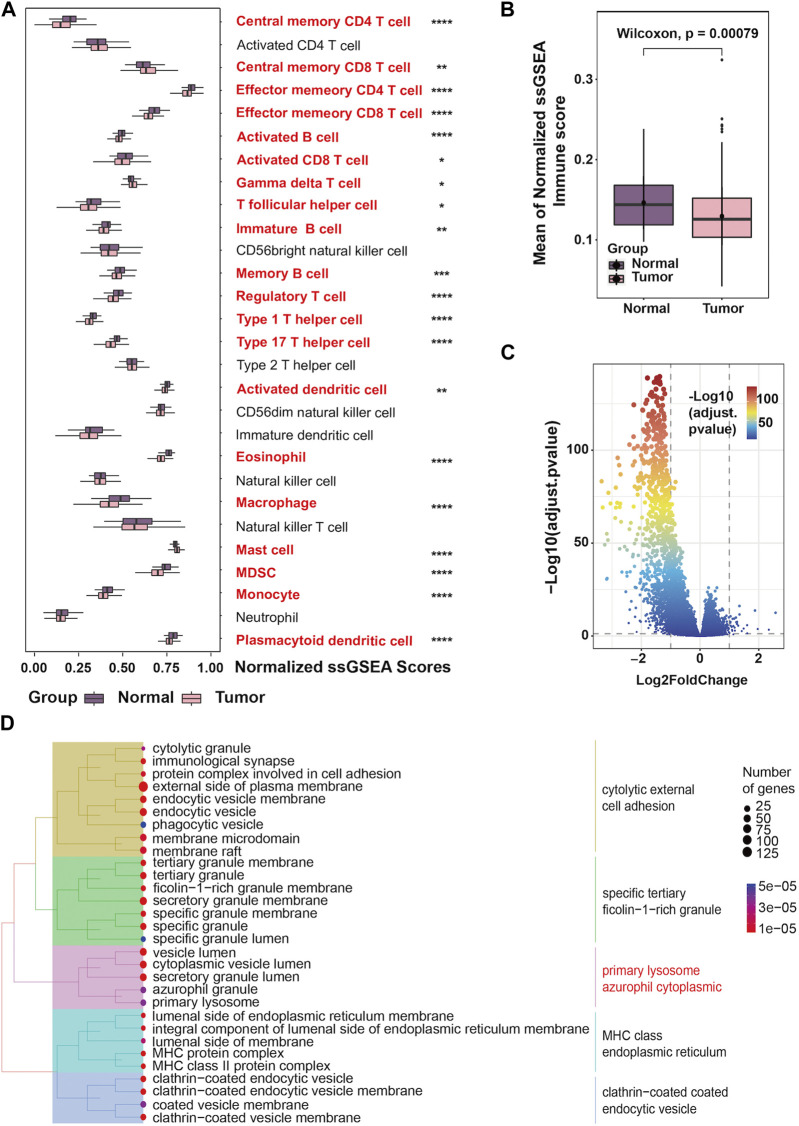
Landscape of the tumor immune microenvironment in prostate cancer **(A)** The difference in the enrichment score of 28 immune cell types between normal and tumor samples; **(B)** Boxplots showing overall immune scores between normal and tumor samples; **(C)** Volcano plot exhibiting DEGs based on immune score classification; **(D)** Tree plots of the GO enrichment analysis of DEGs based on immune score classification.

We obtained data from 500 prostate cancer samples and 52 normal samples from the TCGA-PRAD cohort. Demographics, clinical characteristics and progression status are presented in Table 1. Compared to the non-progression group, mean age is higher in progression group. Besides, the Gleason score shows the most significant difference between the two groups by univariate comparisons of the demographic and clinical characteristics. To assess the unique immune cell infiltration of prostate cancer, which is distinct from normal tissues, we applied the ssGSEA algorithm based on the RNA-sequencing data. The ssGSEA score of 28 immune-related cells in both normal and tumor samples is presented in Figure 1A. The results show that the infiltration of most immune cells (20/28, 71.4%), especially most subtypes of T cells, macrophages, mast cells, plasmacytoid dendritic cells, monocytes and myeloid-derived suppressor cells (MDSC), was significantly different between the normal and tumor samples (*p* < 0.05), which had high potential relativity between the immune microenvironment and prostate cancer. Additionally, the boxplot demonstrated that prostate cancer samples had a lower ssGSEA score, indicating a suppressed immune environment in prostate cancer (Figure 1B). Prostate cancer samples (n = 500) were thereafter divided into two immune subgroups based on the median ssGSEA score; the DEGs are listed in Supplementary Table S3. Compared to the high-immune group, the mRNA expression of most genes in the low-immune group was downregulated (Figure 1C). Of note, these differentially expressed genes in two immune subgroups were considered as IRGs (n = 1,093). The GO enrichment analysis of 1,093 IRGs is shown in Figure 1D. After clustering the GO cellular component enriched pathways, some could be classified into the primary lysosome azurophil cytoplasmic, indicating that some of the IRG products are located in lysosomes or are related to lysosomes when they perform their function.

**TABLE 1:** Demographics, clinical characteristics and progression status.

	Non-Progression (N = 331)	Progression (N = 69)	*p-value*	Test
Age(mean ± SD)	60.61 ± 6.952	61.8 ± 6.242	0.2234	Mann–Whitney test
Race				
Asian	6 (1.81%)	3 (4.35%)	0.6412	Fisher’s exact test
Black or african american	42 (12.69%)	7 (10.14%)		
White	272 (82.18%)	57 (82.61%)		
American indian or alaska native	1 (0.30%)	0 (0.00%)		
Other	10 (3.02%)	2 (2.90%)		
Gleason score				
6	31 (9.37%)	1 (1.45%)	<0.0001	Fisher’s exact test
7	191 (57.7%)	18 (26.09%)		
8	40 (12.08%)	10 (14.49%)		
9	67 (20.24%)	39 (56.52%)		
10	2 (0.60%)	1 (1.45%)		

Abbreviation: SD, standard deviation

### 3.2 Identification of LIRGs between high and low-immune groups and their prognostic relevance

**FIGURE 2:**
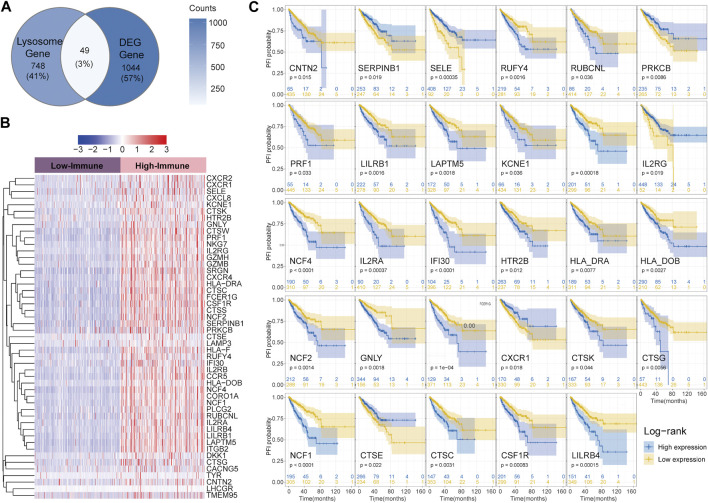
Identification of LIRGs between high and low-immune groups and its prognostic relevance **(A)** Venn diagram showing shared genes between IRGs and LRGs; **(B)** Heatmap of 49 LIRGs; **(C)** PFI analysis of 29 LIRGs associated with the prognosis.

As shown in the Venn diagram, a total of 49 (49/1841, 3%) shared genes between IRGs and LRGs were screened out and regarded as LIRGs (Figure 2A). Figure 2B demonstrates the 49 LIRG expression patterns between two immune subgroups. To assess the prognostic relevance of the 49 candidate LIRGs, we utilized the KM method and found that more than half of the LIRGs (29/49, 59.2%) were associated with the prognosis (all *p* < 0.05) as shown in Figure 2C. As we can see, the high expression of most LIRGs (23/29) predicted a poor prognosis.

### 3.3 Construction of two LIRG prognostic signatures based on gleason score as a risk model in the training set

**FIGURE 3:**
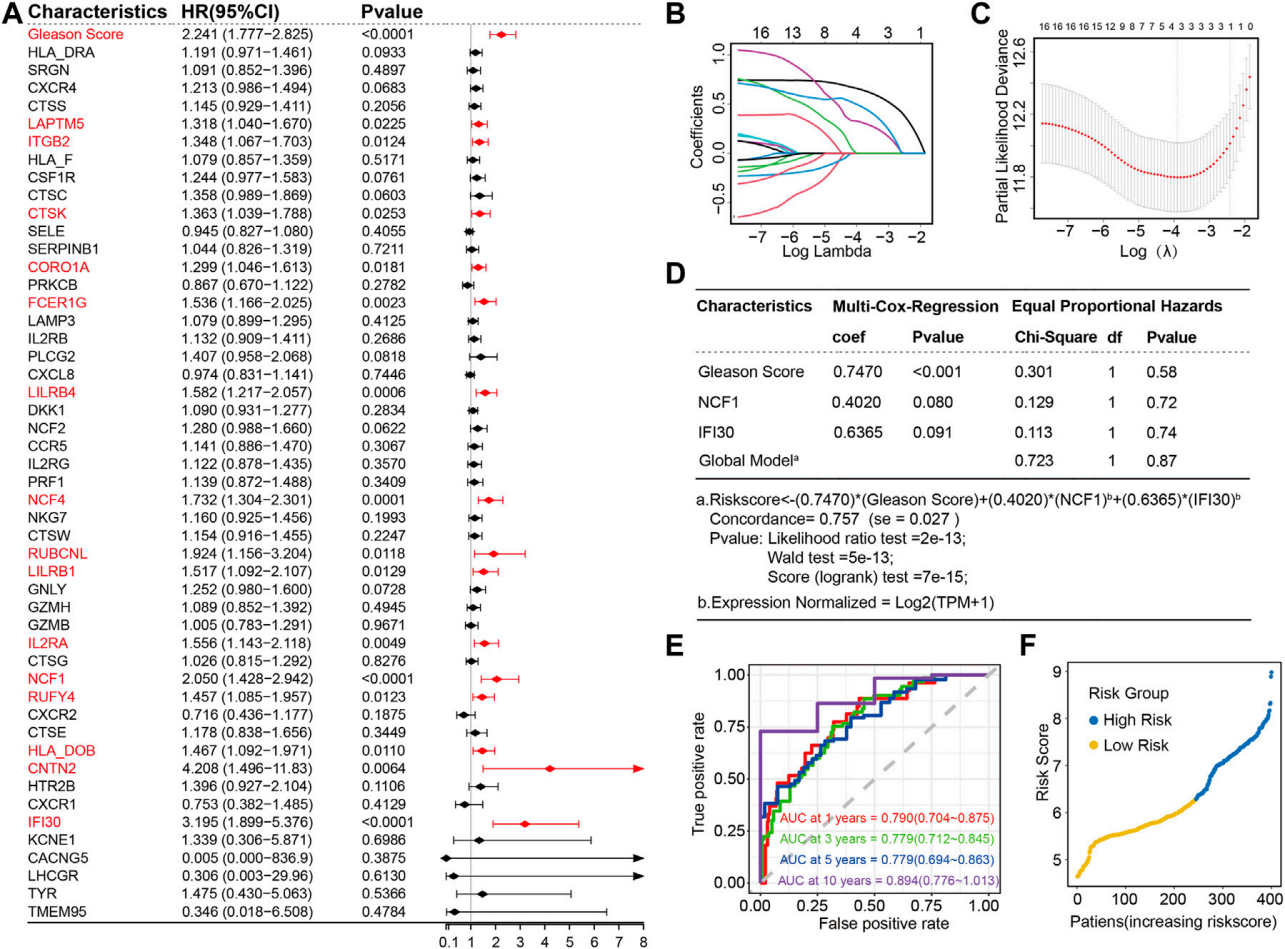
Construction of two LIRG prognostic signatures based on Gleason score as a risk model in the training set **(A)** Forest plot of the Gleason score and 49 candidate LIRGs ex-pression by a univariate Cox regression analysis; **(B)** Lasso regression of the 49 candidate LIRGs; **(C)** Cross-validation in the LASSO regression; **(D)** Multivariable Cox regression analysis of the selected factors; **(E)** Time-dependent ROC curves for the prognostic signatures of 1, 3, 5 and 10-year PFI of PRAD in the training set; **(F)** Distribution of the risk score.

A univariate Cox regression analysis was performed with the Gleason score and the expression profiles of 49 candidate LIRGs in the training set (Figure 3A). In addition to the Gleason score, 15 candidate LIRGs were found to have a significant correlation with progression free interval (all *p* < 0.05, marked in red); the baseline is shown in Supplementary Table S4. Of note, these 16 prognostic signatures were risk factors (HR > 1). To further narrow down candidate prognosis signatures, three candidates, including Gleason score (coefficient = 0.6509428), NCF1 (coefficient = 0.3190647) and IFI30 (coefficient = 0.4826147), were identified based on the optimum λ value 0.02010609 using a LASSO regression analysis (Figures 3B, C). Then, a multivariate Cox regression analysis further screened out the prognosis-associated signature and established a linear risk score formula. As shown in Figure 3D, the risk score was calculated as follows:

$$\left.{Risk\;score=0.7470\times Gleason\;score+0.4020\times {\mathrm{Log}}_{2}\left({TPM}^{NCF1}+1\right)+0.6365\times {\mathrm{Log}}_{2}(TPM}^{IFI30}+1\right)$$

To evaluate the performance of the predictive model constructed by using the risk score, four time-dependent ROC curves were drawn, where the AUC values at 1, 3, 5 and 10 years were 0.790, 0.779, 0.779 and 0.894, respectively (Figure 3E), indicating excellent predictive ability. Patients with prostate cancer were subsequently divided into high and low-risk groups based on the risk score of 6.275728; the cutoff value of the risk score was determined by the ROC analysis (Figure 3F).

**FIGURE 4:**
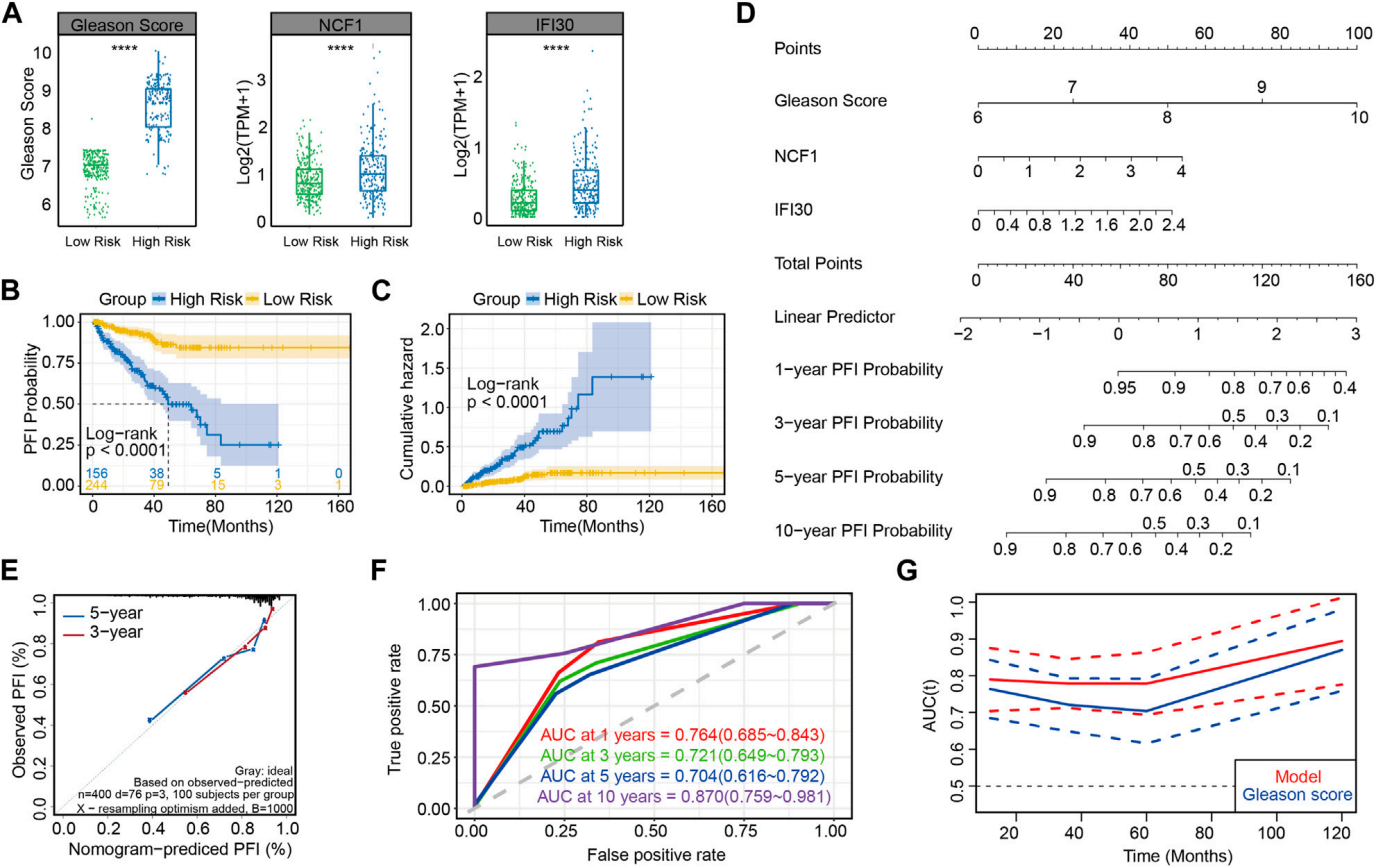
Accuracy for predicting PCa prognosis by using this risk model in the training set **(A)** Gleason scores and the expression of NCF1 and IFI30 in different risk groups; **(B)** PFI curves between two risk groups in the training set; **(C)** Cumulative hazard analysis of two risk groups in the training set; **(D)** Nomogram for predicting the progression-free rate of PRAD patients based on the novel signature; **(E)** Calibration curve showing that the predictive PFI fit the actual PFI well at 3 and 5 years in the training set; **(F)** ROC curves showing the PFI prediction of the novel signature *versus* the Gleason score; **(G)** Plot of AUC curve and its confidence interval at different time.

As shown in the box plot, Gleason scores increased and the expression of NCF1 and IFI30 were upregulated, along with the increasing risk score (Figure 4A). On the basis of the progression free interval analysis, patients in the high-risk group were substantiated to have poorer prognoses by the log-rank test (*p* < 0.0001; Figure 4B). Likewise, in the cumulative hazard analysis, patients with higher risks showed a higher cumulative hazard (Figure 4C). Meanwhile, in order to demonstrate the evaluation of patients’ PFI probability, the nomogram model was established by combining the three prognosis-associated features mentioned above (Figure 4D). Moreover, both 3- and 5-year PFI predictions by nomogram were highly consistent with the actual observation of patients with prostate cancer, as confirmed in the calibration plots (Figure 4E). These results verify that this model, constructed by three prognosis-associated features, is predictive of poor prognoses. Interestingly, the AUC of this risk model was greater than that of the model for which only the Gleason score was considered, whether at 1, 3, 5 or 10 years (Figures 4F, G), suggesting that NCF1 and IFI30 can refine the abilities of prognostic prediction by using the Gleason score alone, which has been the most well-accepted and strongest prognostic predictive tool in prostate cancer.

### 3.4 Validation of prediction of the risk prognostic signature

**FIGURE 5:**
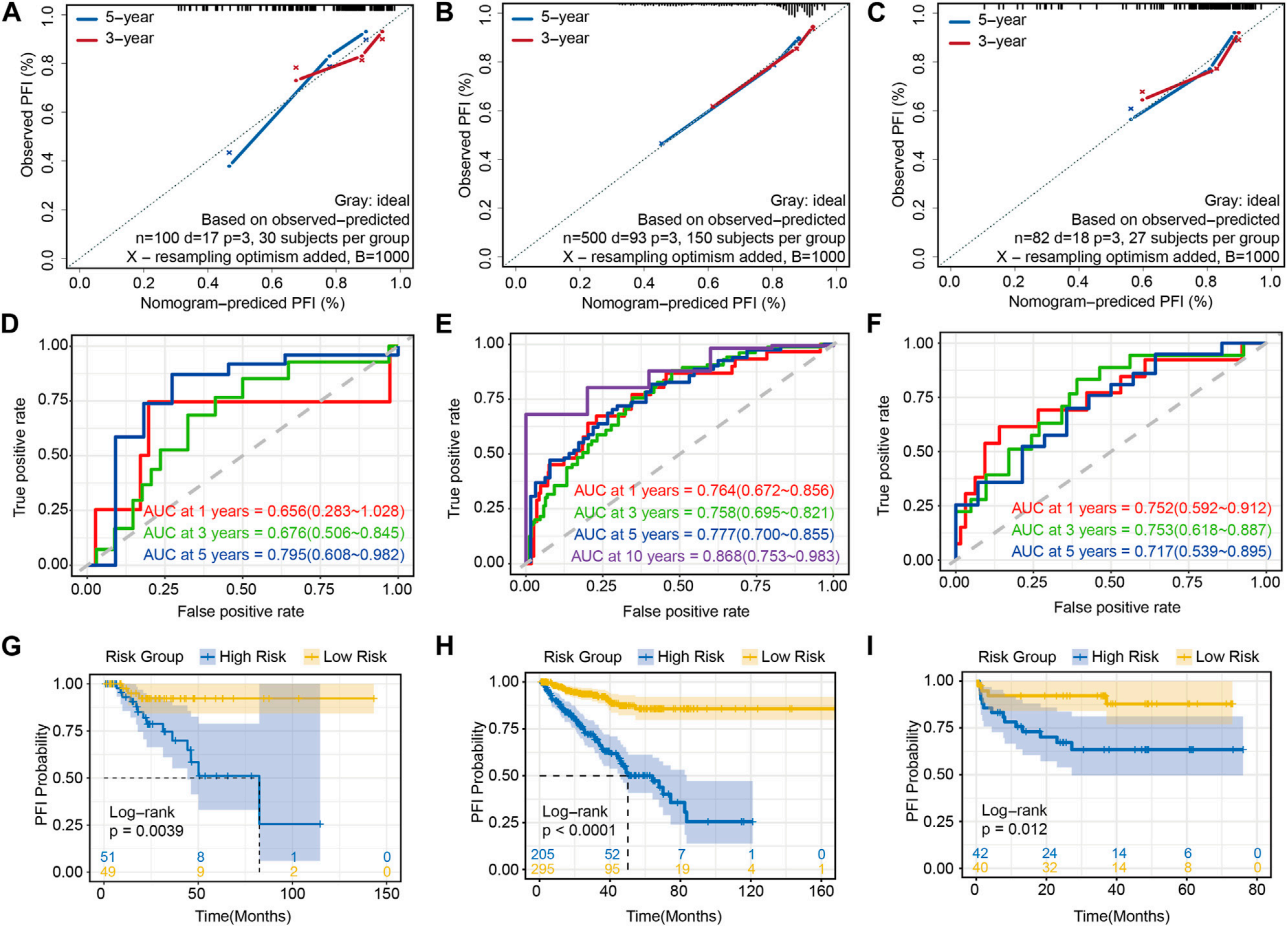
Validations of prediction of the risk prognostic signature: calibration curve showing that the predictive PFI fit the actual PFI well at 3 and 5 years in **(A)** the internal validation set, **(B)** the whole TCGA-PRAD and **(C)** the external validation set. ROC curves showing the PFI prediction of the risk model in **(D)** the internal validation set, **(E)** the whole TCGA-PRAD and **(F)** the external validation set. Univariate regression analysis of the risk score in **(G)** the internal validation set, **(H)** the whole TCGA-PRAD and **(I)** the external validation set.

As mentioned in the Methods section, except for the training set, the remaining 100 cases from TCGA-PRAD were used as the internal validation set (Supplementary Table S5), while 82 cases from EGAS00001002923 were used as the external validation set (Supplementary Table S6). In addition, we also considered the entire TCGA-PRAD database (*n* = 500) as a kind of data cohort for the internal validation set (Supplementary Table S7). First, the calibration curves based on three validation sets showed good agreement between the actual and predicted 3- and 5-year PFI, which illustrated that the risk model was an excellent predictor (Figures 5A–C). Further analysis of AUC by ROC curves ranged from 0.658 to 0.812 (Figures 5D–F), and proved the robust predictive capacity of our model. Meanwhile, a univariate regression analysis was also used to verify the prognostic role of risk score in prostate cancer (Figures 5G–I), revealing that patients with a higher risk score had a poor prognosis.

## 4 Discussion

Lysosomes are a key component of the inner membrane system involved in various biological cell processes, including intracellular transport (Rink et al., 2005), autophagy (Itakura et al., 2012; Yu et al., 2018), metabolic pool (Polishchuk et al., 2014; Lyu et al., 2020), cell proliferation (Bar-Peled et al., 2013), cell migration/adhesion (Lawrence and Zoncu, 2019) and gene expression regulation (Eskelinen, 2006; Ballabio, 2016), as well as having robust prospects as novel anticancer therapeutics, given their emerging role in cancer development and progression. The role of lysosome-related pathways in tumorigenesis and progression of PCa are bidirectional (Levine and Kroemer, 2019). For instance, Wac, an activator of autophagy, heterozygous deletion leads to an increase in cancer progression in PCa, whereas its complete loss constrains it. In other words, it suppresses tumor initiation and promotes the growth of a formed cancer (de la Rosa et al., 2017). Despite the emerging role of lysosomes known in cancer development and progression, its precise effect in predicting PCa outcomes is still unclear.

In this study, we screened out two genes, NCF1 and IFI30, associated with lysosome-related pathways, and illustrated their prognostic role in PCa based on immunoscore classification. Unlike former prognosis predictions, our risk model, consisting of these two genes and the Gleason score, yielded better performance during clinical prognosis prediction than the Gleason score alone. As the Gleason score was defined as an independent hazard factor, NCF1 and IFI30 were identified as risk-related factors. Kaplan–Meier curves substantiated the finding that patients with a higher risk score had a worse PCa outcome.

NCF1, used to denote NOXO2 and p47^PHOX^, is a cytosolic subunit of the NADPH oxidase 2 (NOX2) complex and is associated with reactive oxygen species (ROS) production (Zhao et al., 2017). It has also been documented as exhibiting the capability of functional ROS induction and IL-1 β signaling, and thus enhancing lung colonization of B16F10 melanoma cells (Zhong et al., 2021). Likewise, in our study, patients with higher NCF1 expression showed worse prognoses in PRAD. However, previous studies have revealed that autophagy can be suppressed by interfering with autophagosome-lysosome fusion and lysosomal proteolytic activity *via* the NCF1-ROS axis (Zheng et al., 2016). Meanwhile, NCF1 also played critical roles in multiple immune-associated pathways [1; 2], thereby influenced functional activities of many immune cells. It is reported that NCF1 may positively correlate with pro-tumor M2 macrophages by promoting macrophages differentiating toward M2 macrophages [8]. Natural killer cells and T cells, the major fighters in anti-tumor immune, are prone to apoptosis upon NCF1 acting [4]. NCF1 can also dampen adaptive immunity through preventing the maturation of DCs [5]. Based on previous studies, we speculate that NCF1 might be a promising marker for guiding immunotherapy. However, there is no research indirectly or directly illustrating the correlation between NCF1 and PCa. IFI30, also known as GILT, is the only enzyme known to reduce protein disulfide bonds in the endocytic pathway (Rausch and Hastings, 2015). Besides, the most well-known function of IFI30 is considered to be the promotion MHC class II-restricted processing and presentation in antigens, which is essential for the activation of CD4^+^ T lymphocytes (Phan et al., 2000; Hastings and Cresswell, 2011; West and Cresswell, 2013). Modulation of IFI30 expression to break off the pathway for MHC-restricted tumor antigen presentation has been proven to contribute to malignant cells evading T-cell surveillance of the immune system (Seliger et al., 2000; Haque et al., 2002). Further studies have verified that IFI30 functions as an indispensable factor in the immune response of cancers, such as glioma (Chen et al., 2019), melanoma (Buetow et al., 2019) and breast cancer (Fan et al., 2021, 30). In prostate cancer, IFI30 expression has been confirmed to be associated with its progression, which is consistent with our results (Bao et al., 2011). Non-etheless, its regulatory pathway and biological function have not been well reported. Herein, we elucidated the point that IFI30 may play a predictive role in PCa prognosis *via* the lysosome-immune pathway.

Following a comparison of the ROC curve and nomogram model, our model showed high predictive accuracy and robustness in internal and external validation sets besides the training set. Accordingly, these two LIRGs, coupled with the Gleason score, play an indispensable role as potential biomarkers for predicting PCa outcomes.

There are also several limitations in this study. The data included in this study are all from a public database. That means that more information needs to be collected from more prospective clinical data to prove its practicality. Additionally, more research on the specific mechanisms of this model in PCa is required to clarify *in vitro* and *in vivo* verifications.

## 5 Conclusion

We collectively first identified novel LIRG signatures coupled with the Gleason score to calculate the risk score in PCa, which could measure its risk stratification and predict prognoses. Our results may provide a novel insight on the mechanism of LIRGs in PCa initiation and progression, and offer more robust biomarkers for therapeutic targets for PCa.

## Data Availability

The datasets presented in this study can be found in online repositories. The names of the repository/repositories and accession number(s) can be found in the article/Supplementary Material.
